# Acute postoperative endophthalmitis by *Gemella haemolysans*

**DOI:** 10.4103/0301-4738.62658

**Published:** 2010

**Authors:** Suma Nalamada, Subhadra Jalali, Ashok Kumar Reddy

**Affiliations:** Jhaveri Microbiology Centre, Hyderabad, India; 1 Smt. Kanuri Santhamma Vitreoretinal Centre, Kallam Anji Reddy Campus, L.V. Prasad Eye Institute, Hyderabad, India

**Keywords:** Endophthalmitis, *Gemella haemolysans*, intraocular vancomycin

## Abstract

Endophthalmitis is a rare and serious post-surgical complication. We report a case of acute postoperative endophthalmitis after an uneventful cataract surgery caused by a commensal organism, *Gemella haemolysans*. The patient was successfully treated with vitrectomy and intravitreal antibiotics like vancomycin, along with topical cefazolin.

*Gemella haemolysans* are aerobic or facultative anaerobic, gram-positive cocci which easily decolorize and appear as gram-negative diplococci. Because of this characteristic, the organism was originally classified under the genus *Neisseria.* Based on chemotaxomic studies, it was subsequently placed in a distinct genus, *Gemella* within the family *Streptococcacea.*[1,2] It is a normal commensal organism found in the oral cavity and upper respiratory tract. It has been implicated in a variety of human infections like meningitis, septic arthritis, osteomyelitis, thoracic empyema with lung abscess, and endocarditis.[3] We report a case of endophthalmitis caused by *G. haemolysans*. We believe it is the first reported case of this rare species from ocular infection from our country.

## Case Report

A 60-year-old non-diabetic, hypertensive man was seen one day after onset of decreased vision associated with severe watering, eyelid swelling and pain. Symptoms had started four days (February 2009) after uneventful phacoemulsification with intraocular lens implantation surgery for age-related cataract in the left eye.

On examination, the left eye visual acuity was light perception with accurate projection of rays. Slit-lamp biomicroscopy of the pseudophakic eye showed lid edema, congested conjunctiva, microcystic corneal edema, no corneal infiltrates, 1.0 mm hypopyon, 3+ cells and flare in anterior chamber and fixed dilated pupil.[4] The intraocular pressure (IOP) was 31 mmHg done by applanation tonometry. Fundus examination showed vitreous exudates. On ocular ultrasonography, the retina appeared attached while low to medium reflective membranes with fine after-movements were noted in the vitreous cavity. Apparently the right eye examination was normal.

Acute pseudophakic postoperative endophthalmitis was diagnosed in the left eye. After informed consent, patient underwent a standard undiluted vitreous biopsy, pars plana vitrectomy and intraocular antibiotics were instilled; the antibiotics were vancomycin 1.0 mgm, ceftazidime 2.25 mg and dexamethasone 400 μgm. Postoperative topical medication included 3% ciprofloxacin and 1% prednisolone acetate eye drops every one hour, 1% homatropine eye drops four times a day and 0.5% timolol maleate eye drops and oral ciprofloxacin 750 mg twice a day.

The vitreous biopsy specimen was processed in the microbiology laboratory as per the institutional protocol.[5] Direct microscopic examination of vitreous sample revealed gram-positive cocci in pairs, short chains and tetrads. A confluent growth of gram-positive cocci with alpha haemolysis on blood agar was observed after 24 h of incubation. The organism was further identified as *Gemella haemolysans* using Mini API Rapid ID 32 Strep. The organism was sensitive to cefazolin, gentamicin, gatifloxacin, chloramphenicol, and vancomycin and resistant to amikacin, ciprofloxacin, ofloxacin and moxifloxacin.

On the eighth day, the antibiotic regimen was changed to 5% fortified cefazolin (10 ml of distilled water added to 500 ml cefazolin injection vial) eye drops one-hourly for three weeks and oral chloramphenicol 250 mg three times a day for a week. Four weeks after surgery his best corrected visual acuity improved to 20/80 N 12 and IOP was 16 mmHg. All medication was discontinued and topical steroids were tapered over next four weeks.

## Discussion

*Gemella* species were first isolated from the throat, nose, and eyes in measles patients by Tunicliff in 1917.[6] They are infrequently isolated from clinical specimens and are often confused with viridans-type streptococci. Identification is difficult. A literature search revealed only four reported cases of various eye infections caused by *Gemella haemolysans.*[7–10]

Munir and Kailasanathan *et al*., reported *Gemella haemolysans* as a causative organism for infectious crystalline keratopathy. In both cases the organism was identified by automated systems API Rapid strep and complete resolution of the disease occurred with topical vancomycin therapy. The third case, reported by Ritterband *et al.,* had a history of sarcoidosis and was on long-term systemic prednisolone. The patient developed bacterial keratitis and consecutive endophthalmitis. Surgical intervention with keratoplasty, pars plana vitrectomy, and intravitreal antibiotics led to resolution of the infection. The endophthalmitis case reported by Raman *et al.,*[10] was similar to our case of infection with a rare commensal organism in a healthy, immunocompetent individual. The postoperative cases, our case and the one reported by Raman *et al.*, did not have any corneal involvement and presented with acute onset of vitreous exudates and hypopyon.

*Gemella* species are usually susceptible to a wide variety of antimicrobial agents including penicillin, ampicillin, rifampicin, and vancomycin but some isolates have demonstrated low-level resistance to aminoglycosides and trimethoprim.[11] Our isolate of *Gemella* spp. was susceptible to cefazolin, vancomycin, chloramphenicol, gatifloxacin and resistant to amikacin, ofloxacin and ciprofloxacin. There is only one case of vancomycin resistance reported in literature and our case showed good response to intravitreal vancomycin.[12] Postoperatively, our patient received topical and oral ciprofloxacin that was changed to topical fortified cefazolin and oral chloramphenicol, based on *in vitro* susceptibility tests. The patient showed rapid resolution of symptoms and signs of infection in follow-up. Kailasanathan *et al.,* also observed rapid recovery in signs and symptoms after changing the antibiotics.

To conclude, as diagnostic technology improves, more and more rare organisms especially those that are commensal organisms or low-virulence organisms, will be identified as causing acute postoperative endophthalmitis. With good microbiological evaluation including drug sensitivity and timely intervention with appropriate medication results appear promising as seen in our case.
